# Mild drought conditions at the tillering stage promote dry matter accumulation and increase grain weight in drip-irrigated spring wheat (*Triticum aestivum* L.)

**DOI:** 10.3389/fpls.2024.1509325

**Published:** 2025-01-06

**Authors:** Yilin Ma, Jingyi Cai, Shuting Bie, Ziqiang Che, Guiying Jiang, Jianguo Liu

**Affiliations:** College of Agriculture, Shihezi University, Shihezi, China

**Keywords:** spring wheat, drip irrigation, assimilate transport, grouting character, drought and re-watering during fertility periods

## Abstract

**Introduction:**

In order to elucidate the physiological mechanism of post-flowering assimilate transport regulating the formation of yields in arid regions and to provide technological support for further water-saving and high yields in the wheat region in Xinjiang, we conducted a study on the effects of different fertility periods and different degrees of drought and re-watering on the post-flowering dry matter accumulation and transport of spring wheat and the characteristics of grain filling.

**Methods:**

In two spring wheat growing seasons in 2023 and 2024, a split-zone design was used, with the drought-sensitive variety Xinchun 22 (XC22) and drought-tolerant variety Xinchun 6 (XC6) as the main zones and a fully irrigated control during the reproductive period [CK, 75%~80% field capacity (FC)], with mild drought at the tillering stage (T1, 60%~65% FC), moderate drought at the tillering stage (T2, 45%~50% FC), mild drought at the jointing stage (J1, 60%~65% FC), and mild drought at the jointing stage (J2, 45%~50% FC) as the sub-zones.

**Results:**

The dry matter accumulation of the aboveground parts of wheat (stem sheaths, leaves, and spikes), the transfer rate and contribution rate of nutrient organs, the maximum filling rate (V_max_), and the mean filling rate (V_mean_) increased significantly after re-watering in the T1 treatment, and decreased with the deepening of the degree of water stress. The 13C isotope tracer results also showed that the T1 treatment increased the distribution rate of 13C assimilates in the grain at maturity. Correlation and principal component analyses showed that grain weight was highly significantly and positively correlated with stem sheath, leaf, and spike dry matter accumulation, amount of nutrient organ post-flowering transports, transport rate, contribution rate, the onset and the termination time of the rapid growth period, V_max_, and V_mean_, and stem sheath and spike dry matter accumulation had a direct effect on grain weight. While the two varieties performed differently among the treatments, both exhibited optimal performance in the T1 treatment.

**Discussion:**

In conclusion, mild drought at the tillering stage (60%-65% FC) was the best model for water conservation and high yield of wheat under the conditions of this trial.

## 1 Introduction

Utilizing a crop’s own regulatory capacity, tapping its physiological water-saving potential, and achieving efficient crop water use through drip irrigation under the premise of high and stable yield are important ways to guarantee food security and ecological security in Xinjiang and achieve sustainable agricultural development (Geng et al., 2021). Wheat is the most important grain crop in Xinjiang. Drip irrigation is currently the main cultivation technology for wheat production in Xinjiang. The application and development of drip irrigation water-saving technology over the years have been fully exploited for crop yield and water use (Umair et al., 2019). Previous studies have found that moderate drought stress during the reproductive period of crops is directly related to plant morphogenesis, biomass accumulation, and yield formation in the middle and late stages, resulting in compensatory or even supercompensatory effects (Ru et al., 2024a, Ru et al., 2024b; Abid et al., 2016a). Therefore, the use of wheat’s own regulatory capacity and the compensatory effect formed in the process of “drought re-watering” to coordinate the efficient transfer of post-flowering assimilates to the grain and achieve water conservation and high yields has become the key to the efficient production of wheat in Xinjiang.

Coordinating the efficient transport of photosynthetic assimilates to the grain is a scientific challenge in cereal crops. It is of great theoretical and practical significance to elucidate the mechanisms and pathways for promoting the transfer of assimilates to kernels in cereal crops, and to solve the problems related to low assimilate transfer rate to kernels in the nutrient organs, slow grain filling, and poor grain filling in wheat production in arid zones under the current high level of water supply. Wheat yield formation is mainly due to the direct post-flowering translocation of assimilates to the grain (Zhang et al., 2023; Matsuura and An, 2020). Approximately 80% of the yield in high-yielding wheat comes from the accumulation and translocation of dry matter after flowering (Ali et al., 2018). Allahverdiyev and Huseynova (2017) found that drought stress causes adaptive changes in the distribution of dry matter among stems, leaves, and spikes of wheat after flowering, accelerating the reduction of stem and leaf dry matter. Plaut et al. (2004) showed that a water deficit significantly reduced the rate of dry matter accumulation in grains and decreased stem and leaf dry weights during filling. The impact of drought stress on crops is closely associated with factors such as the timing, intensity, and duration of the stress experienced (Ozturk et al., 2022; Cui et al., 2019). Drought training can enable crops to develop drought-resistant memories, allowing them to better withstand future adversity (Wu and Yang, 2024; Zhao et al., 2020). Drought training in wheat during the nutrient growth stage is beneficial as it weakens the adverse effects of a post-flowering drought (Ning et al., 2023). Furthermore, the positive effect of maintaining a field capacity of 55%-60% during the tillering stage on sustaining high yields is significantly greater than that observed during the jointing stage (Cui et al., 2019). Ma et al. (2015) found that deficit irrigation at the tillering stage significantly increased pre-flowering dry matter remobilization and post-flowering accumulation of dry matter in wheat, but deficit irrigation at the booting stage negatively affected pre-flowering dry matter remobilization and post-flowering accumulation of dry matter, which were significantly lower compared to controls. Liu et al. (2015) found that mild drought during the jointing stage, characterized by a field capacity of 65%-70%, improved pre-flowering canopy structure, sustained high post-flowering photosynthesis levels, and enhanced grain filling. However, under moderate drought conditions, with a field capacity of 55%-60%, the accumulation of post-flowering dry matter in wheat decreased, which adversely affected its transport to the grains and ultimately resulted in reduced yield. In arid regions, the effects of drought and re-watering during the early and middle stages of growth on the accumulation of dry matter in the leaves, stems, and sheaths of spring wheat, and its efficient transport to the grains, remain unclear and require further research.

The efficient transport of assimilates to grains following flowering is essential for enhancing crop yield. Grain-filling is a dynamic process, in which the rate and duration of filling are critical parameters that influence filling characteristics, which are closely associated with grain weight (Li et al., 2022; Lv et al., 2017a). Previous studies have indicated a positive correlation between the duration of grain filling and grain weight, while the relationship between grain-filling rate and grain weight is not significant (Yang et al., 2008). Some studies suggest that both grain filling rate and filling duration influence grain weight, with the former having a greater effect (Dias and Lidon, 2009). Water plays a crucial role in determining the grain filling process and the thousand grain weight of wheat (Prado et al., 2023). Abid et al. (2016b) found that under severe drought conditions during the jointing stage (with a field capacity of 35%-40%), wheat exhibited an accelerated grain filling rate, a shortened filling duration, and a reduction in grain weight after flowering. Liu et al. (2016) found that there were significant differences in the response of different varieties and their superior grains and inferior grains to soil moisture. Moderate drought conditions significantly promoted wheat grain filling and increased maximum and average grain filling rates.

Drip irrigation technology has become the main cultivation technology for wheat production in Xinjiang, realizing the integration of water and fertilizer with intelligent field management and reducing labor and input costs (Chen et al., 2021; Wang et al., 2020). Currently, research on the effects of drought on dry matter accumulation after flowering and grain-filling characteristics primarily emphasizes the impact of sustained drought stress (Hnilička et al., 2007). However, there is a notable lack of systematic studies investigating the effects of drought rehydration on the dry matter accumulation after flowering, transport in various organs, and grain-filling characteristics of spring wheat across different growth stages and degrees in arid regions. This study analyzed the accumulation and transport of dry matter after flowering under different stages and degrees of drought stress, clarified the relationship between grain weight and characteristic parameters of grain filling, and elucidated the regulatory mechanism that promotes the transport of assimilates to grains after flowering. The results provide a scientific basis for water conservation and a high wheat yield in arid areas.

## 2 Materials and methods

### 2.1 Experimental site and design

**Figure 1:**
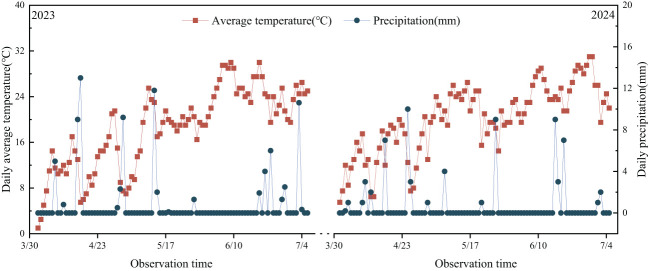
Daily rainfall and average daily temperature during the two experimental seasons in 2023 and 2024.

The experiment was implemented from April 2023 to July 2024 at the Agricultural College Experimental Station of Shihezi University in Shihezi City, Xinjiang (44°18’N, 85°59’E). The multi-year average temperature in Shihezi was 7.8°C. The multi-year average precipitation was 208 mm and the multi-year average evaporation was 1200 mm, with a relative humidity of approximately 65%. The meteorological parameters during the growth period are shown in **Figure 1**. According to the meteorological data, the average temperature during the wheat cultivation period was 18.5°C in 2023 and 20.2°C in 2024. Similarly, the sum precipitation during the cultivation periods was 82 mm and 65 mm in 2023 and 2024, respectively. The tested soil type was irrigated gray desert soil. The basic characteristics of the 0-60 cm soil tested are shown in **Table 1**.

**Table 1:** Basic properties of the 0-60 cm soil for testing.

Year	Total nitrogen content/ (g·kg^-1^)	Available hydrolysis nitrogen content/ (mg·kg^-1^)	Available phosphorus content/ (mg·kg^-1^)	Available potassium content/ (mg·kg^-1^)	Organic matter content/ (g·kg^-1^)	pH
2023	1.28	55.75	15.89	132.04	17.82	7.6
2024	1.30	55.65	15.83	132.01	18.34	7.7

The trial was conducted in a split-zone design with three repetitions, with the spring wheat (*Triticum aestivum* L.) varieties as the main plots. The varieties for testing were the main cultivars ‘Xinchun 22’ (drought-sensitive type, XC22) and ‘Xinchun 6’ (drought-tolerant type, XC6) in Xinjiang (Zhang et al., 2024; Jing et al., 2023). The moisture treatments were subzones, and a total of five moisture treatments were set up: normal irrigation during the entire growth period (CK), mild drought at the tillering stage (T1), moderate drought at the tillering stage (T2), mild drought at the jointing stage (J1), and moderate drought at the jointing stage (J2) (**Table 2**). To ensure that the two drought rehydration times were the same, the moderate drought was water-controlled five days earlier than the mild drought treatment, and the drought treatment was held for 10 days after the corresponding water control amount was reached, with rapid irrigation at the end of the treatment to bring it back to the control level (referred to as re-watering).

**Table 2:** Experimental set-up for limited irrigation during the reproductive period of drip-irrigated spring wheat.

Treatment	Tillering stage	Jointing stage
CK	75%-80% FC	75%-80% FC
T1	60%-65% FC	75%-80% FC
T2	45%-50% FC	75%-80% FC
J1	75%-80% FC	60%-65% FC
J2	75%-80% FC	45%-50% FC

FC is the field water capacity.

**Figure 2:**
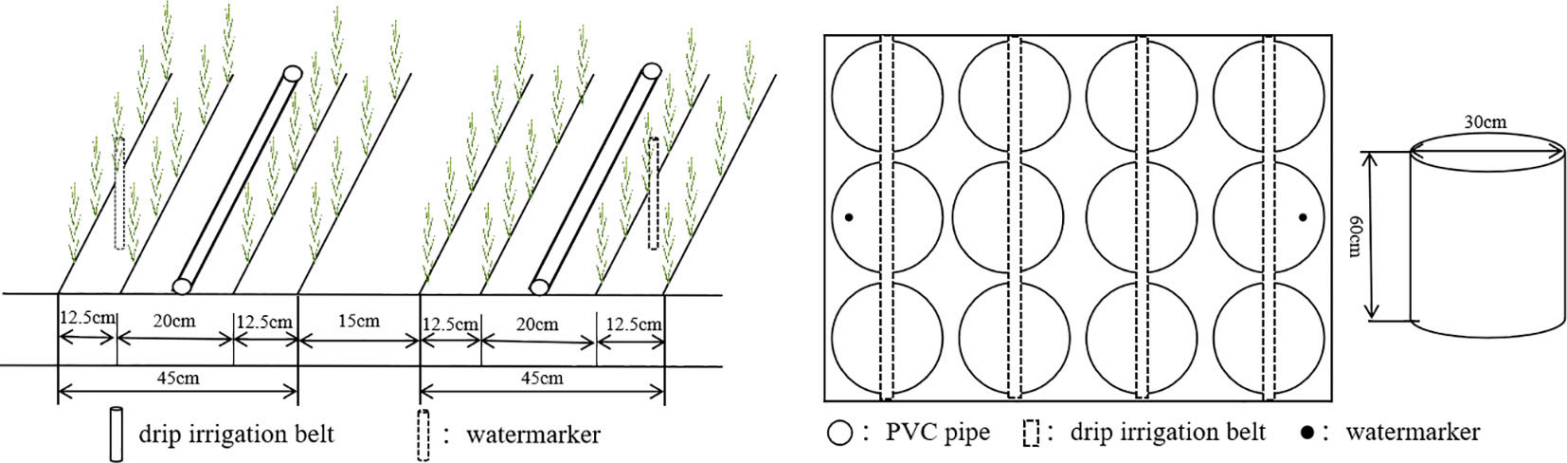
Soil column cultivation and field community layout in 2023 and 2024.

A soil column cultivation trial and a field plot validation trial were set up (**Figure 2**), both with a sowing rate of 345 kg·hm^-2^. Unsealed PVC pipes (diameter of 30 cm, thickness of 1 cm, and length of 60 cm) were used for the soil column cultivation trial. A 60 cm deep square soil pit was dug in each plot and 60 cm long PVC pipes were put into the pit in an orderly manner. Soil from the 0-20 cm, 20-40 cm, and 40-60 cm layers was taken out and sieved after drying and then placed into the PVC pipe in order. The soil was filled until 40 cm deep and then fully filled with water to make it sink naturally. When it was completely settled, 5 cm of topsoil was then filled in with water to settle it down. Basal fertilizer was applied according to the area of the pipe and this was finally covered with 15 cm of topsoil. The soil around the PVC pipe was filled in so that the upper portion of the PVC pipe was flush with the ground. Drip irrigation tape (16 mm pipe diameter, 30 cm drip head spacing, and 2.6 L·h^-1^ flow rate) was laid in the upper part of the PVC pipe (through the center) for irrigation. A soil column cultivation trial is mainly used for indicator determination.

Field plot trials were conducted with plot sizes of 12 m^2^ (3 m × 4 m). One m^2^ was equivalent to approximately 525 wheat plants. The plants were planted in wide and narrow rows (12.5 + 20 + 12.5 + 15 cm) in a “one tube, four rows” formation, and the drip irrigation tape was placed in the 20 cm wide rows. A 60 cm impermeable membrane was buried between the plots to prevent water migration. Nitrogen was applied at 255 kg·hm^-2^ during the growing period, and the ratio of basal to topdressing nitrogen fertilizer was 3:7. Before sowing, 120 kg·hm^-2^ of P_2_O_5_ (superphosphate, P_2_O_5_ = 12%) and 30% of nitrogen fertilizer (urea, N=46%) were plowed into the soil as the basal fertilizer. Furthermore, 70% of the nitrogen fertilizer was first dissolved in the fertilizer tank at a ratio of 20:40:35:5 during the tillering stage, jointing stage, booting stage, and filling stage, and then applied to the soil with water. Irrigation was controlled by a water meter. The seeds were sown on 3 April 2023 and 1 April 2024 and harvested on 7 July 2023 and 5 July 2024, respectively. Due to the small size of the trial site, field management (mainly pest and weed control) was done manually. The field plot trial was mainly used to measure wheat yield at maturity and to support the soil column cultivation trial.

**Figure 3:**
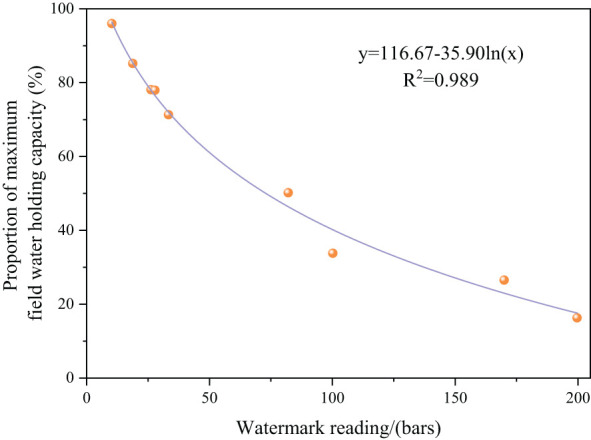
Ratio of soil water content to maximum field capacity and Watermark reading curve.


We utilized a Watermark resistive water tension sensor (model 200SS; Irrometer Co., Riverside, USA) to monitor variations in soil moisture content. The Watermark sensor was positioned vertically in the soil at a depth of 20 cm in the middle of a 12.5 cm wheat row within the community. The Watermark readings were recorded daily at 20:00 throughout the wheat growth period. Prior to the experiment, a 0-20 cm soil layer was extracted from the field for the measurement of field moisture content. The Watermark values were then used to derive a fitting curve that illustrated the relationship between soil moisture and the maximum field water holding capacity (**Figure 3**). Current soil moisture content was calculated based on this curve. The amount of irrigation required was determined using the following formula (Wang et al., 2015).

$$m=10\rho_{b}H(\beta_{I}-\beta_{j})$$

In the formula, m is the irrigation amount (mm); *ρ_b_* is the soil bulk density; *H* is the planned depth (cm); *β_I_* is the target moisture content (field capacity multiplied by the target relative moisture content); and *β_j_* is the current soil water content.

### 2.2 Measurement items and methods

#### 2.2.1 Dry matter accumulation after anthesis

At the flowering stage, wheat plants that had bloomed on the same day and had essentially the same growth were selected and tagged as sampling and observation materials, and 10 plants were randomly selected from each plot at 7, 14, 21, 28, and 35 days after flowering for measurement. This was repeated the times. Each wheat plant was divided into three parts, ie., stem sheaths, leaves, and spikes, and each part was placed in an oven at 105°C for 30 min for blanching, dried at 80°C until constant weight, and weighed after cooling to calculate dry matter accumulation

The post-flowering dry matter transport amount (DMTA) was calculated according to Formula 1 (Han et al., 2010):


(1) $$\text{DMTA}\left(\text{g}\cdot {\text{hm}}^{-2}\right)=\text{N}1-\text{N}2$$

In the formula, N1 is the dry matter amount in the flowering stage (g·hm^-2^) and N2 is the dry matter amount at the maturity stage (g·hm^-2^).

The post-flowering dry matter transport rate (DMTR) was calculated according to Formula 2 (Arduini et al., 2006):


(2) DMTR(%)=(DMTA/N1)×100%

In the formula, DMTA is the post-flowering dry matter transport amount and N1 is the dry matter amount (g·hm-2) in the flowering stage. The post-flowering dry matter contribution rate (DMCR) was calculated according to Formula 3 (Dordas, 2009):


(3) DMCR(%)=(DMTA/N3)×100%

In the formula, DMTA is the post-flowering dry matter transport amount and N3 is the grain yield (g·hm^-2^) at the flowering stage.

#### 2.2.2 Grain-filling characteristics

At the flowering stage, wheat plants that had bloomed on the same day and had essentially the same growth were selected and tagged to serve as sampling and observation material. Samples were taken at 7-day intervals from the 7th day after flowering until wheat maturity. In total, 30 main stem spikes were randomly selected from each plot for threshing and bagging. Afterward, they were placed in an oven at 105°C for 30 min for blanching, dried at 80°C until constant weight, and weighed after cooling. This was repeated three times.

Logistic growth curves were used to fit the grain weight gain process using grain weight (Y) as the dependent variable and days after flowering (T) as the independent variable (4) (Yan et al., 2022):


(4) $$Y=k/\left(1+ae^{-bT}\right)$$

In the formula, Y is grain weight, T is days after flowering, k is maximum growth, and a and b are constants. The results were analyzed to obtain the onset time (t_1_), termination time (t_2_), the duration (t_2_-t_1_) of the rapid growth period, the time to maximum filling rate (T_max_=lnb/k), the mean filling rate (V_mean_=kb/lna+4.45512), and the maximum filling rate (V_max_=ak/4)

#### 2.2.3 ^13^C isotope

At the flowering stage, wheat plants that had bloomed on the same day and had essentially the same growth were selected and tagged to serve as sampling and observation material. On a morning with sunny weather at the flowering stage, the whole flag leaf of the wheat plant was covered and sealed with a sealing bag. Then, 3.5 ml of ^13^CO_2_ (99 atom % ^13^C, Shanghai Research Institute of Chemical Industry, China) was injected into the bag with a syringe and the pinhole was immediately sealed with tape. After 30 min of photosynthetic reaction, the ^13^CO_2_ remaining in the bag was recovered by NaOH solution and the sealing bag was removed. Samples were taken at 72h after labeling and at maturity. Three plants were sampled each time and this was repeated three times. Samples at 72h were divided into three parts: leaf, stem sheath, and spike rachis + glumes, and samples at maturity were divided into four parts: leaf, stem sheath, spike rachis + glumes, and grain, which were dried to a constant weight and ground. The δ^13^C content was determined using an Isoprime 100 Stable Isotope Ratio Mass Spectrometer (Isoprime Ltd., UK) coupled with a vario MICRO cube Elemental Analyser (Elementar Ltd., Germany) and further calculated by the following (Ge et al., 2012):


(5) $$\begin{array}{l} F_{\text{i}}(\%)=\frac{({\text{δ}}^{13}C+1000)\times R_{PDB}}{({\text{δ}}^{13}C+1000)\times R_{PDB}+1000}\times 100 \end{array}$$

In the formula, F_i_ is the ^13^C abundance and R_PDB_ (standard ratio of carbon isotopes) = 0.0112372.

^13^C accumulation in each organ (g·hm^-2^) = (labelled sample F_i_ - unlabelled sample F_i_) × total organ mass (g·hm^-2^) × organ total carbon content (%).

^13^C distribution rate of each organ (%) = ^13^C accumulation in single organ (g·hm^-2^)/total ^13^C accumulation in single stem (g·hm^-2^) × 100%.

#### 2.2.4 Yield

At maturity, 1 m^2^ of wheat plants were selected from each plot and harvested manually. The wheat spike number per unit area was determined and 20 spikes of wheat were randomly selected from them and the grain per spike was determined. All the harvested wheat ears were threshed, dried, and weighed to determine the thousand grain weight and calculate the yield. The is repeated three times.

### 2.3 Data analysis

We used SPSS 26.0 to analyze the data. Multiple comparisons were conducted between different treatments using the minimum significant difference test (*P*<0.05) (n=3). Images were plotted using OriginLab 2021 (Northampton, MA, USA). Correlation plots and principal component analysis (PCA) plots were drawn using OriginLab 2021 (Northampton, MA, USA) (n=3).

## 3 Results

### 3.1 Dry matter accumulation and distribution after anthesis

#### 3.1.1 Dry matter accumulation in stem sheaths

**Figure 4:**
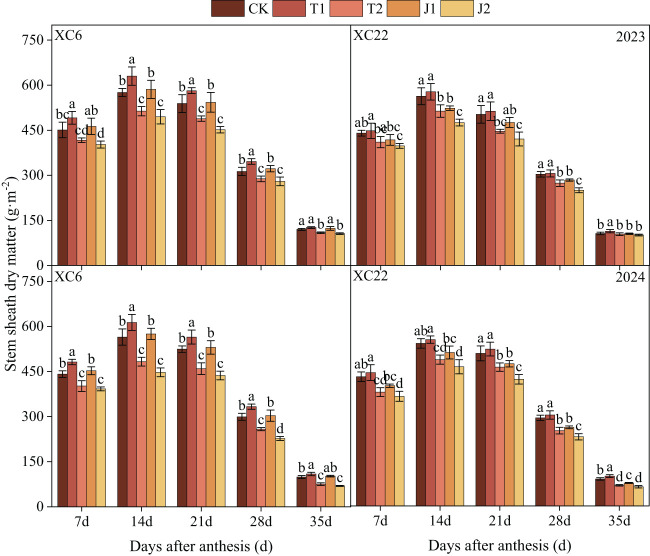
Effect of drought and re-watering on stem sheath dry matter accumulation in drip-irrigated spring wheat at different growth periods. XC6, Xinchun 6; XC22, Xinchun 22; CK, normal irrigation during the growth stage; T1, mild drought during the tillering stage; T2, moderate drought during the tillering stage; J1, mild drought during the jointing stage; J2, moderate drought during the jointing stage. Different lowercase letters indicate that different treatments of the same variety have significant differences at the 0.05 level.

Stem sheath dry matter accumulation in both varieties of drip-irrigated spring wheat showed an increasing and then decreasing trend with days after flowering, both reaching a maximum at 14d after flowering and then continuing to decline (**Figure 4**). Stem sheath dry matter accumulation was significantly affected by drought stress (**Table 3**). It also decreased with increasing degree of stress and the drought treatment at the tillering stage was better than the drought treatment at the nodulation stage. In both the T1 and J1 treatments of XC6, stem sheath dry matter was able to recover to the CK level, and the T1 treatment showed a greater compensatory effect than the J1 treatment. The stem sheath dry matter accumulation under the T1 treatment was on average 6.31%~38.10% higher than the other treatments, and the J1 treatment was on average 15.17%~31.52% higher than J2. In XC22, only the stem sheath dry matter under the T1 treatment recovered to the CK level. Stem sheath dry matter accumulation under the T1 treatment was on average 2.50%~33.88% higher than in other treatments and 7.32%~13.65% higher in the J1 than in the J2 treatment. The stem sheath dry matter accumulation of XC6 was higher than that of XC22 at all periods, with the stem sheath dry matter accumulation of XC6 under the T1 treatment being significantly higher than that of XC22 by an average of 9.93% at 14d after flowering.

**Table 3:** Analysis of variance of dry matter accumulation and grain weight in drip-irrigated spring wheat by drought and re-watering at different fertility periods.

Trait	Stem sheath dry matter					Leaf dry matter				
	7d	14d	21d	28d	35d	7d	14d	21d	28d	35d
V	**	**	**	**	*	**	**	**	ns	**
T	**	**	**	**	**	**	**	**	**	**
V×T	*	*	*	ns	ns	ns	**	*	ns	ns

Trait	Spike dry matter					Grain weight				
	7d	14d	21d	28d	35d	7d	14d	21d	28d	35d
V	**	**	**	**	**	**	**	**	*	ns
T	**	**	**	**	**	**	**	**	**	**
V×T	ns	ns	ns	ns	ns	ns	ns	ns	ns	ns

T, treatment; V, variety; * and ** indicate significant differences at the 0.05 and 0.01 levels, respectively; ns indicates no significant difference.

#### 3.1.2 Dry matter accumulation in leaves

**Figure 5:**
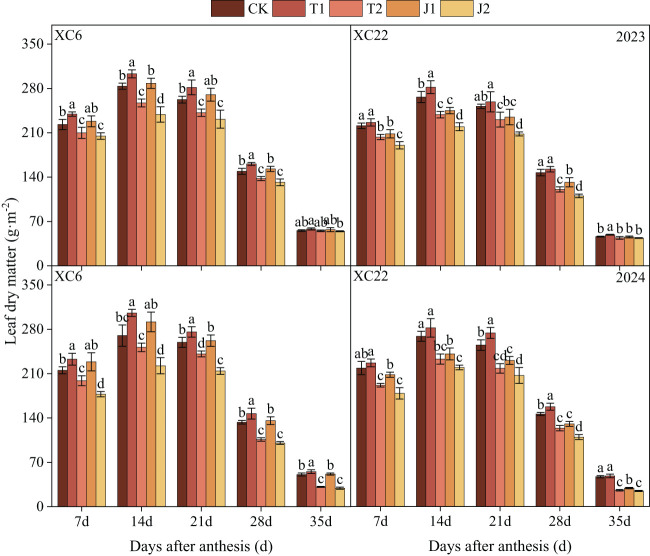
Effect of drought and re-watering on leaf dry matter accumulation in drip-irrigated spring wheat at different growth periods. XC6, Xinchun 6; XC22, Xinchun 22; CK, normal irrigation during the growth stage; T1, mild drought during the tillering stage, T2, moderate drought during the tillering stage; J1, mild drought during jointing stage; J2, moderate drought during the jointing stage. Different lowercase letters indicate that different treatments of the same variety have significant differences at the 0.05 level.

With the progression of the fertility process, the leaf dry matter accumulation of both varieties of drip-irrigated spring wheat showed a tendency to increase and then decrease, and all of them reached a maximum at 14d after flowering (**Figure 5**). In the same period, all treatments performed optimally under the T1 treatment. Leaf dry matter accumulation was significantly affected by drought stress (**Table 3**). Leaf dry matter accumulation decreased with an increased degree of stress, and the drought treatment at the tillering stage was superior to the drought treatment at the jointing stage. Leaf dry matter accumulation in the two varieties behaved differently. Leaf dry matter accumulation of XC6 was restored to the CK level under both the T1 and J1 treatments, and the T1 treatment showed a greater compensatory effect. Leaf dry matter accumulation under the T1 treatment was on average 3.41%~48.17% higher than the other treatments, and dry matter accumulation under the J1 treatment was on average 19.52%~40.77% higher than the J2 treatment. The leaf dry matter accumulation of XC22 recovered to the CK level only in the T1 treatment, which was 3.05%~54.37% higher than the other treatments on average. Leaf dry matter accumulation under the J1 treatment was on average 10.65%~19.22% higher than that of the J2 treatment. Leaf dry matter accumulation of XC6 was higher than that of XC22 in all periods and the leaf dry matter accumulation of XC6 under the T1 treatment was significantly higher than that of XC22 by 7.96% at 14d after anthesis.

#### 3.1.3 Dry matter accumulation in spikes

**Figure 6:**
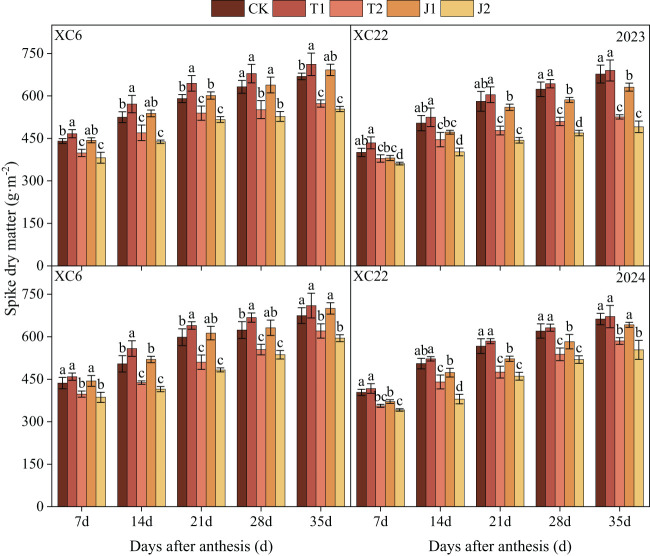
Effect of drought and re-watering on spike dry matter accumulation in drip-irrigated spring wheat at different growth periods. XC6, Xinchun 6; XC22, Xinchun 22; CK, normal irrigation during the growth stage; T1, mild drought during the tillering stage; T2, moderate drought during the tillering stage; J1, mild drought during the jointing stage; J2, moderate drought during the jointing stage. Different lowercase letters indicate that different treatments of the same variety have significant differences at the 0.05 level.

With the advancement of the reproductive process, the spike dry matter accumulation of drip-irrigated spring wheat of both varieties showed a gradually increasing trend and reached a maximum value at 35d after flowering (**Figure 6**). Drought stress had a significant effect on spike dry matter accumulation (**Table 3**) and with an increase in the degree of stress, the spike dry matter accumulation decreased significantly. There were differences in the performance of the two varieties in terms of spike dry matter accumulation. Spike dry matter accumulation under both the T1 and J1 treatments of XC6 recovered to the CK level, and T1 showed a greater compensatory effect than the J1 treatment. Spike dry matter accumulation under the T1 treatment was on average 4.30%~23.90% higher than the other treatments, and under the J1 treatment, it was on average 15.53%~24.01% higher than the J2 treatment. In XC22, only the accumulation of spike dry matter under the T1 treatment was able to recover to the CK level and was on average 5.93%~30.86% higher than the other treatments. When comparing the varieties, the spike dry matter accumulation of XC6 was higher than that of XC22 in all periods, in which the spike dry matter accumulation of XC6 under the T1 treatment was 4.45% higher than that of XC22 at 35d after anthesis.

#### 3.1.4 Dry matter transfer rate and contribution rate

The effects of moisture treatments on the DMTA (68.79g·m^-2^~259.61g·m^-2^), DMTR (14.65%~52.41%), and DMCR (13.34%~41.28%) of wheat nutrient organs post-flowering reached significant levels (**Table 4**). With increasing drought, the DMTA, DMTR, and DMCR of all nutrient organs post-flowering decreased. The DMTA, DMTR, and DMCR of the stem sheaths of XC6 post-flowering under the T1 treatment were on average 42.55%, 31.76%, and 22.40% higher than that of the T2 treatment, respectively, while the DMTA, DMTR, and DMCR of the stem sheaths of XC6 post-flowering under the J1 treatment were on average 42.98%, 31.45%, and 23.02% higher than that of the J2 treatment, respectively. The DMTA, DMTR, and DMCR of the stem sheaths of XC22 post-flowering under the T1 treatment were on average 77.46%, 64.31%, and 45.50% higher than that of the T2 treatment, and the DMTA, DMTR, and DMCR of the stem sheaths of XC22 post-flowering under the J1 treatment were 80.02%, 67.97%, and 47.50% higher than that of the J2 treatment, respectively. There were differences in the performance of the two varieties after re-watering under all treatments. The DMTR and DMCR of the nutrient organs of XC6 post-flowering in the T1 and J1 treatments were restored to the CK level and the compensatory effect of T1 was greater than that of the J1 treatment. The DMTR and DMCR of the leaves post-flowering in the T1 treatment were on average 12.51% and 9.07% higher than in the CK treatment. In XC22, only the DMTR and DMCR of the nutrient organs post-flowering in the T1 treatment recovered to the CK level. The DMTR and DMCR of the leaves post-flowering in the T1 treatment were on average 3.53% and 2.87% higher than that of the CK treatment. Both varieties of wheat exhibited higher DMTRs and DMCRs in stem sheaths than in leaves post-flowering. The DMTR and DMCR of the stem sheaths post-flowering were on average 30.24% and 31.00% higher than that of the leaves in the T1 treatment for XC6, while the DMTR and DMCR of the stem sheaths post-flowering were on average 23.13% and 26.60% higher than that of the leaves in the T1 treatment for XC22. The post-flowering DMTR and DMCR of the leaves of XC6 under the T1 treatment were on average 11.75% and 9.42% higher than that of XC22. Variety reached a significant level for the post-flowering DMTA, DMTR, and DMCR of each nutrient organ. The moisture treatments reached highly significant levels for the post-flowering DMTA, DMTR, and DMCR of each nutrient organ. Variety and moisture treatments had significant reciprocal effects on the post-flowering DMTA, DMTR, and DMCR of the stem sheaths.

**Table 4:** Effects of drought and re-watering on dry matter transport and contribution rate to nutrient organs in drip-irrigated spring wheat at different fertility periods.

Year (Y)	Variety (V)	Treatment (T)	DMTA post-flowering(g·m^-2^)		DMTR post-flowering (%)		DMCR post-flowering (%)	
			Stem sheaths	Leaves	Stem sheaths	Leaves	Stem sheaths	Leaves
2023	XC6	CK	221.32 ± 10.68 b	159.73 ± 26.04 bc	44.88 ± 0.10 b	32.53 ± 4.77 bc	37.24 ± 0.22 b	26.77 ± 2.85 bc
		T1	259.61 ± 3.07 a	195.71 ± 22.16 a	52.41 ± 2.32 a	39.74 ± 3.82 a	41.28 ± 1.35 a	31.03 ± 2.07 a
		T2	159.39 ± 18.12 c	128.49 ± 13.11 cd	35.06 ± 3.40 c	29.76 ± 2.25 c	31.53 ± 2.12 c	25.43 ± 1.42 c
		J1	238.25 ± 6.38 ab	177.09 ± 11.05 ab	48.08 ± 0.80 b	36.05 ± 1.20 ab	39.01 ± 0.36 ab	28.97 ± 0.75 ab
		J2	154.80 ± 15.59 c	119.77 ± 16.15 d	34.76 ± 2.52 c	27.92 ± 3.11 c	31.31 ± 1.53 c	24.20 ± 1.99 c
	XC22	CK	221.83 ± 11.88 a	167.53 ± 13.38 a	45.46 ± 4.63 a	34.78 ± 4.33 a	36.69 ± 2.49 a	27.72 ± 2.61 a
		T1	231.23 ± 8.95 a	179.10 ± 6.16 a	46.25 ± 1.06 a	36.83 ± 2.59 a	37.46 ± 0.75 a	29.04 ± 1.52 a
		T2	121.64 ± 14.31 c	101.70 ± 23.27 c	27.45 ± 2.07 c	25.24 ± 5.33 b	26.41 ± 1.48 c	21.98 ± 3.63 b
		J1	179.90 ± 4.69 b	126.27 ± 3.50 b	36.83 ± 0.40 b	26.23 ± 1.52 b	31.97 ± 0.26 b	22.45 ± 1.01 b
		J2	104.59 ± 8.49 c	85.91 ± 2.17 c	24.47 ± 1.53 c	22.13 ± 0.12 b	24.27 ± 1.20 c	19.96 ± 0.12 b
	F	V	**	**	**	*	**	*
		T	**	**	**	**	**	**
		V×T	*	ns	*	ns	*	ns
2024	XC6	CK	215.47 ± 5.06 ab	170.17 ± 0.40 a	44.53 ± 0.47 ab	35.39 ± 1.27 ab	35.88 ± 0.15 a	28.34 ± 0.71 a
		T1	236.74 ± 1.77 a	183.40 ± 9.02 a	46.81 ± 1.48 a	36.40 ± 3.71 a	37.36 ± 0.67 a	28.97 ± 2.14 a
		T2	193.68 ± 14.05 bc	142.51 ± 2.80 b	41.05 ± 1.97 b	29.80 ± 0.58 bc	32.81 ± 1.20 b	24.17 ± 0.38 b
		J1	226.93 ± 3.24 a	177.56 ± 17.40 a	45.42 ± 2.06 a	35.79 ± 5.43 a	36.31 ± 1.10 a	28.44 ± 3.22 a
		J2	171.84 ± 22.31 c	131.37 ± 12.13 b	36.46 ± 4.13 c	27.86 ± 1.51 c	29.90 ± 2.37 c	22.89 ± 0.97 b
	XC22	CK	184.44 ± 14.84 a	151.43 ± 12.05 a	37.15 ± 1.57 a	31.13 ± 1.24 a	31.20 ± 0.95 a	25.62 ± 0.76 a
		T1	191.47 ± 12.07 a	154.51 ± 18.22 a	38.00 ± 0.19 a	31.49 ± 2.32 a	32.13 ± 0.06 a	25.87 ± 1.44 a
		T2	116.17 ± 13.57 b	82.19 ± 11.57 b	23.73 ± 4.00 b	17.10 ± 3.20 b	21.54 ± 2.96 b	15.24 ± 2.46 b
		J1	178.49 ± 9.38 a	146.39 ± 3.76 a	36.29 ± 3.62 a	30.61 ± 0.09 a	30.01 ± 2.24 a	24.58 ± 0.07 a
		J2	94.92 ± 4.72 c	68.79 ± 11.72 b	19.57 ± 1.72 b	14.65 ± 3.13 b	18.38 ± 1.28 b	13.34 ± 2.52 b
	F	V	**	**	**	**	**	**
		T	**	**	**	**	**	**
		V×T	*	*	*	*	*	*

XC6, Xinchun 6; XC22, Xinchun 22; Y, year; T, treatment; V, variety; DMTA, dry matter transport amount; DMTR, dry matter transport rate; DMCR, dry matter contribution rate. Different lowercase letters indicate that different treatments of the same variety have significant differences at the 0.05 level. * and ** indicate significant differences at the 0.05 and 0.01 levels, respectively; ns indicates no significant difference.

#### 3.1.5 ^13^C assimilate accumulation and distribution rate

The ^13^C isotopes were labelled in the T1 and CK treatments of different varieties of drip-irrigated spring wheat (**Table 5**). At 72h after labelling and at maturity, the ^13^C assimilate accumulation and distribution rate showed different performance. In the different varieties, the highest ^13^C assimilate accumulation and distribution rate in the stem sheaths were found 72h after labelling, and the highest ^13^C assimilate accumulation and distribution rate in the grain were found at maturity. At 72h after labeling and at maturity, the accumulation of ^13^C assimilates in leaves, stem sheaths, and glumes+cobs in the T1 treatment was higher than that of the CK treatment. At 72h after labelling, the stem sheath accumulation of XC6 in the T1 treatment was significantly higher than that in the CK treatment by 18.96%; at maturity, the stem sheath accumulation of XC6 in the T1 treatment was significantly higher than that in the CK treatment by 7.86%, which was the same as that of the dry matter accumulation and translocation pattern. At maturity, the grain distribution rate of XC6 under the T1 treatment was 3.92% higher than that of XC22.

**Table 5:** Effect of drought and re-watering on ^13^C assimilate accumulation and distribution rate in drip-irrigated spring wheat at different fertility periods.

Stage	Variety	Treatment	Accumulation (g·hm^-2^)					Distribution rate (%)			
			Total	Leaves	Stem sheaths	Spike rachis + glumes	Grain	Leaves	Stem sheaths	Spike rachis + glumes	Grain
72h	XC6	CK	448.35 ± 17.05b	66.03 ± 4.59b	274.26 ± 6.00b	108.06 ± 6.48b	—	14.72 ± 0.46b	61.20 ± 0.97ab	24.09 ± 0.52b	—
		T1	551.64 ± 26.97a	85.44 ± 4.26a	326.27 ± 17.72a	139.93 ± 5.00a	—	15.49 ± 0.02a	59.14 ± 0.31c	25.38 ± 0.33a	—
	XC22	CK	412.39 ± 23.21b	60.23 ± 2.92b	254.09 ± 12.97b	98.07 ± 7.51b	—	14.61 ± 0.24b	61.63 ± 0.45a	23.76 ± 0.48b	—
		T1	422.50 ± 14.22b	64.49 ± 1.62b	254.43 ± 10.01b	103.58 ± 2.77b	—	15.27 ± 0.13a	60.21 ± 0.38bc	24.52 ± 0.29b	—
Maturity	XC6	CK	387.68 ± 19.24b	34.94 ± 3.12b	66.83 ± 3.67b	36.05 ± 2.11b	249.86 ± 10.53b	9.00 ± 0.35a	17.24 ± 0.23b	9.30 ± 0.09b	64.47 ± 0.58b
		T1	488.45 ± 24.64a	44.51 ± 4.14a	72.08 ± 2.57a	48.58 ± 3.23a	323.29 ± 14.86a	9.10 ± 0.38a	14.76 ± 0.29c	9.94 ± 0.16a	66.20 ± 0.35a
	XC22	CK	345.57 ± 21.35c	30.60 ± 2.03b	64.00 ± 1.36b	31.00 ± 2.45c	219.97 ± 15.50c	8.85 ± 0.05a	18.55 ± 0.73a	8.96 ± 0.15c	63.63 ± 0.54b
		T1	362.26 ± 20.94bc	32.14 ± 1.85b	65.75 ± 2.67b	33.53 ± 0.96bc	230.84 ± 15.49bc	8.87 ± 0.01a	18.16 ± 0.32a	9.27 ± 0.26bc	63.70 ± 0.59b

XC6, Xinchun 6; XC22, Xinchun 22. Different lowercase letters indicate that different treatments have significant differences at the 0.05 level.

### 3.2 Grain-filling parameters

#### 3.2.1 Grain weight

**Figure 7:**
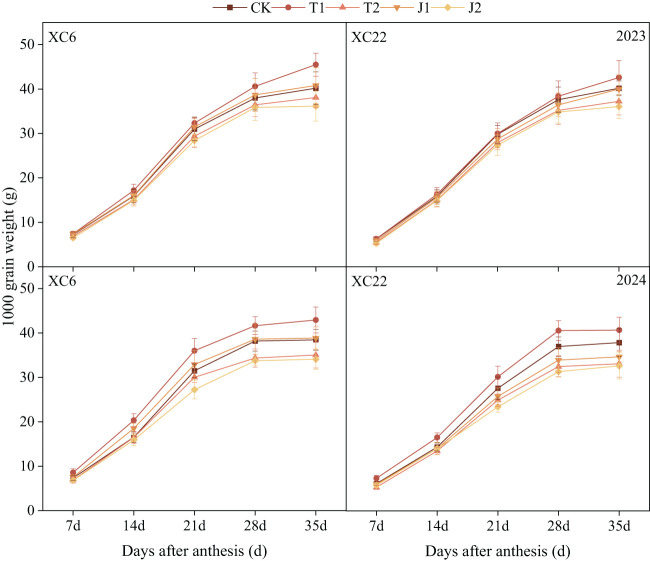
Effects of drought and re-watering on the grain weight of drip-irrigated spring wheat at different growth stages. XC6, Xinchun 6; XC22, Xinchun 22; CK, normal irrigation during the growth stage; T1, mild drought during the tillering stage; T2, moderate drought during the tillering stage; J1, mild drought during the jointing stage; J2, moderate drought during the jointing stage. Different lowercase letters indicate that different treatments of the same variety have significant differences at the 0.05 level.

The grain weight of two varieties peaked at 35d after anthesis, both of which grew faster from 7-28d after anthesis, and the growth trend slowed down from 28-35d after anthesis (**Figure 7**). Grain weight was significantly affected by drought stress (**Table 3**) and decreased with increasing stress levels. In the same period, the grain weight in the T1 treatment was higher than the other treatments. At 35d after anthesis, the grain weights of XC6 and XC22 averaged 44.21g and 41.64g under the T1 treatment. The XC6 grain weight under the T1 treatment was on average 7.05%-25.87% higher than other treatments and 11.16%-15.78% higher under the J1 treatment than the J2 treatment. The grain weight of XC6 was higher than that of XC22 in all periods, with the grain weight of XC6 under the T1 treatment being significantly higher than that of XC22 by an average of 6.14% at 35d after anthesis.

#### 3.2.2 Characteristic parameters of grain filling

The coefficient of determination (R^2^) was above 0.99 when the logistic growth curve equation was used to fit the grain weight variation, indicating that the fitted equations effectively described the grain-filling process (**Table 6**). Both varieties had the highest theoretical thousand grain weight under the T1 treatment. The theoretical thousand grain weight of XC6 was on average 10.79%-24.44% higher than the other treatments under the T1 treatment, and the theoretical thousand grain weight of XC22 was on average 7.33%-21.93% higher than the other treatments. When analyzing the irrigation process, we found that t_1_ in XC6 was at 11-15d after flowering, t_2_ was at 23-30d after flowering, and t_2~_t_1_ was at 12-16d after flowering. Moreover, t_1_ in XC22 was at 12-14d after flowering, t_2_ was at 24-29d after flowering, and t_2_-t_1_ was at 13-16d after flowering. The V_mean_ and V_max_ of XC6 under the T1 treatment increased by an average of 4.59% and 6.12%, respectively, compared to the CK treatment.

**Table 6:** Effect of drought and re-watering on grain-filling parameters in drip-irrigated spring wheat at different fertility periods.

Year	Variety	Treatment	Fitting equation	R^2^	t_1_	t_2_	t_2_-t_1_ (d)	T_max_ (d)	V_mean_ (g/1000 grains)	V_max_ (g/1000 grains)
2023	XC6	CK	y=41.450/(1 + 22.192e^-0.195t^)	0.9969**	12.33	25.81	13.48	15.87	1.75	2.02
		T1	y=47.139/(1 + 19.191e^-0.175t^)	0.9987**	14.50	29.57	15.06	16.90	1.77	2.06
		T2	y=39.443/(1 + 22.582e^-0.196t^)	0.9965**	12.02	25.44	13.42	15.89	1.69	1.93
		J1	y=42.128/(1 + 22.542e^-0.196t^)	0.99636*	12.37	25.81	13.44	15.90	1.78	2.06
		J2	y=37.690/(1 + 24.05e^-0.204t^)	0.99543*	11.36	24.29	12.94	15.62	1.68	1.92
	XC22	CK	y=41.344/(1 + 23.62e^-0.195x^)	0.9996^**^	12.36	25.89	13.53	16.25	1.74	2.01
		T1	y=44.024/(1 + 21.1e^-0.180x^)	0.9997**	13.70	28.31	14.62	16.92	1.72	1.98
		T2	y=38.197/(1 + 25.528e^-0.202x^)	0.9998**	11.53	24.59	13.06	16.06	1.69	1.93
		J1	y=41.095/(1 + 22.38e^-0.187x^)	0.9998**	12.82	26.90	14.08	16.61	1.68	1.92
		J2	y=37.120/(1 + 26.61e^-0.206x^)	0.9994**	11.13	23.89	12.76	15.89	1.68	1.92
2024	XC6	CK	y=39.920/(1 + 23.482e^-0.209t^)	0.9943*	11.33	23.93	12.60	15.09	1.80	2.09
		T1	y=43.871/(1 + 20.128e^-0.21t^)	0.9976**	11.73	24.27	12.54	14.29	1.94	2.30
		T2	y=35.878/(1 + 22.93e^-0.220t^)	0.9959*	10.30	22.29	11.99	14.26	1.72	1.97
		J1	y=39.997/(1 + 22.244e^-0.217t^)	0.9970**	10.94	23.10	12.15	14.31	1.85	2.17
		J2	y=35.434/(1 + 17.884e^-0.195t^)	0.9973**	11.56	25.08	13.52	14.81	1.55	1.73
	XC22	CK	y=39.806/(1 + 25.094e^-0.194t^)	0.9961*	12.22	25.82	13.60	16.64	1.69	1.93
		T1	y=43.063/(1 + 21.321e^-0.189t^)	0.9935*	12.96	26.93	13.96	16.22	1.75	2.03
		T2	y=34.505/(1 + 24.582e^-0.200t^)	0.9974**	11.15	24.34	13.20	16.05	1.54	1.72
		J1	y=36.377/(1 + 21.842e^-0.191t^)	0.9966**	11.95	25.78	13.82	16.19	1.55	1.73
		J2	y=34.376/(1 + 17.11e^-0.175t^)	0.9975**	12.70	27.76	15.06	16.24	1.38	1.50

XC6, Xinchun 6; XC22, Xinchun 22; t_1_, the onset time of the rapid growth period; t_2_, the termination time of the rapid growth period; t_2_-t_1_, the duration time of the rapid growth period; T_max_, the time to maximum filling rate; V_mean_, the mean filling rate; V_max_, the maximum filling rate. Different lowercase letters indicate that different treatments of the same variety have significant differences at the 0.05 level. * and ** indicate significant differences at the 0.05 and 0.01 levels, respectively; ns indicates no significant difference.

### 3.3 Yield

The moisture treatments significantly affected the spike number and thousand grain weight of drip-irrigated spring wheat and had a highly significant effect on yield (**Table 7**). The yield trend and its composition was different for the different varieties. Yield was highest under the T1 treatment and decreased with increased stress. The yield of XC6 under the T1 treatment was on average 18.26% higher than the T2 treatment and 16.99% higher under the J1 treatment than the J2 treatment, respectively; the yield of XC22 was on average 16.52% higher than T2 treatment under T1 treatment and 15.98% higher in J1 treatment than J2 treatment, respectively. Compared with CK, T1 increased grain per spike and thousand grain weight, and the grain per spike and thousand grain weight of XC6 increased by an average of 2.61% and 5.67%, respectively, under the T1 treatment compared with CK. In contrast, the spike number slightly decreased under the tT1 treatment compared with the CK treatment, the spike number of XC6 under the T1 treatment decreased by 4.92% on average compared with the CK treatment, and the spike number of XC22 under the T1 treatment decreased by 4.40% on average.

**Table 7:** Effect of drought and re-watering on yield and its composition in drip-irrigated spring wheat at different fertility periods.

Year (Y)	Variety (V)	Treatment (T)	Spike number /10^4^·hm^−2^	Grain per spike	Thousand grain weight/g	Yiled/kg·hm^-2^
2023	XC6	CK	430.85 ± 17.45a	36.98 ± 1.83a	46.64 ± 2.09ab	6997.25 ± 95.85b
		T1	425.53 ± 10.96ab	37.25 ± 2.09a	48.43 ± 1.56a	7524.65 ± 71.44a
		T2	400.04 ± 13.70b	36.84 ± 2.67a	45.43 ± 2.32ab	6673.54 ± 50.15c
		J1	427.54 ± 15.23ab	36.91 ± 2.53a	47.45 ± 1.63a	7023.52 ± 287.75b
		J2	417.98 ± 16.81ab	35.91 ± 1.43a	43.02 ± 2.69b	6302.67 ± 134.26d
	X22	CK	428.43 ± 16.15a	36.58 ± 2.22ab	46.45 ± 3.26a	7118.29 ± 161.48a
		T1	417.65 ± 19.18ab	36.95 ± 1.71a	47.13 ± 2.41a	7084.42 ± 125.27a
		T2	407.43 ± 10.36ab	34.35 ± 1.59ab	45.23 ± 2.21a	6314.76 ± 52.28c
		J1	424.43 ± 10.49ab	35.21 ± 1.56ab	45.56 ± 1.77a	6664.43 ± 50.83b
		J2	400.01 ± 13.48b	33.42 ± 1.55b	43.93 ± 1.48a	5772.45 ± 255.63d
	F	V	ns	ns	ns	**
		T	*	ns	*	**
		V×T	ns	ns	ns	*
2024	XC6	CK	420.21 ± 10.67a	39.00 ± 0.82b	45.47 ± 0.77ab	6883.35 ± 120.32a
		T1	384.03 ± 10.53b	40.75 ± 0.65a	48.88 ± 0.96a	7364.55 ± 140.16a
		T2	362.48 ± 5.52c	37.68 ± 0.75b	44.42 ± 3.78b	5950.30 ± 478.13b
		J1	406.72 ± 8.72a	38.09 ± 0.66b	48.25 ± 1.31a	6907.02 ± 175.44a
		J2	365.44 ± 4.07c	38.34 ± 0.96b	42.52 ± 0.87b	5636.51 ± 273.50b
	X22	CK	408.48 ± 4.66a	38.50 ± 0.80a	44.52 ± 1.91ab	6992.26 ± 13.36a
		T1	382.82 ± 10.39b	39.22 ± 0.43a	47.66 ± 2.99a	7001.88 ± 282.54a
		T2	361.58 ± 10.75c	36.66 ± 0.49b	43.97 ± 1.23ab	5793.45 ± 334.70c
		J1	405.17 ± 5.75a	38.07 ± 0.94a	44.43 ± 2.66ab	6318.14 ± 348.28b
		J2	359.81 ± 9.08c	36.31 ± 0.69b	42.19 ± 1.94b	5422.79 ± 28.77c
	F	V	ns	*	ns	*
		T	**	**	*	**
		V×T	ns	ns	ns	ns

XC6, Xinchun 6; XC22, Xinchun 22; Y, year; T, treatment; V, variety. Different lowercase letters indicate that different treatments of the same variety have significant differences at the 0.05 level. * and ** indicate significant differences at the 0.05 and 0.01 levels, respectively; ns indicates no significant difference.

### 3.4 Correlation of parameters related to dry matter accumulation and grain filling with yield and grain weight

**Figure 8:**
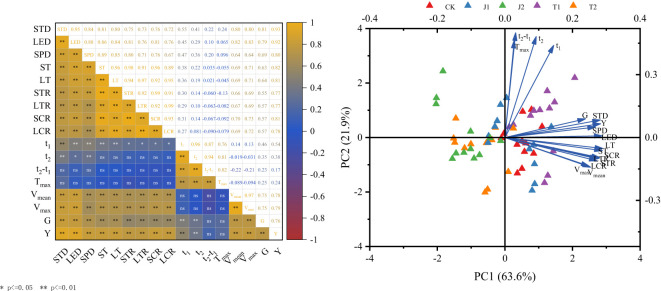
The correlation between drought and re-watering at different growth stages and dry matter accumulation, grain-filling parameters, and grain weight in drip-irrigated spring wheat. G, grain weight; STD, stem sheath dry matter accumulation; LED, leaf dry matter accumulation; SPD, spike dry matter accumulation; ST, stem sheath DMTA; LT, leaf DMTA; STR, stem sheath DMTR; LTR, leaf DMTR; SCR, stem sheath DMCR; LCR, leaf DMCR; t_1_, the onset time of the rapid growth period; t_2_, the termination time of the rapid growth period; t_2_-t_1_, the duration time of the rapid growth period; T_max_, the time to maximum filling rate; V_mean_, the mean filling rate; V_max_, the maximum filling rate. * and ** indicate significant differences at the 0.05 and 0.01 levels, respectively; ns indicates no significant difference; n=3 (repeated 3 times).

The dry matter accumulation and grain filling parameters of spring wheat in the drought and re-watering conditions exhibited varying degrees of correlation with yield and grain weight (**Figure 8**). Yield (Y) and grain weight (G) were highly significantly positively correlated with stem sheath dry matter accumulation (STD), leaf dry matter accumulation (LTD), spike dry matter accumulation (SPD), stem sheath DMTA (ST), stem sheath DMTR (STR), stem sheath DMCR (SCR), leaf DMTA (LT), leaf DMTR (LTR), leaf DMCR (LCR), t_1_, t_2_, (V_mean_) and V_max_ (*r*=0.35**~0.93**), with all of them having a high correlation with STD (*r*=0.81**~0.93**), and Y and G were not significantly correlated with t_2_-t_1_ and T_max_)(*r*=0.17~0.25). Furthermore, STD, LTD, SPD, ST, STR, SCR, LT, LTR, and LCR were highly significantly positively correlated with V_mean_ and V_max_ (*r*=0.64**~0.83**). This showed that the yield and grain weight of spring wheat under drip irrigation under drought and re-watering conditions are closely related to dry matter accumulation and grain filling, and that increasing dry matter accumulation and promoting grain filling are conducive to increasing grain weight and yield.

Principal component analysis further revealed that principal component 1 and principal component 2 contributed 63.6% and 21.9%, respectively, with a cumulative contribution of 85.5%, i.e., 2 principal components explained 85.5% of the individual indicators. Principal component 1 included STD, LTD, SPD, ST, STR, SCR, LT, LTR, LCR, V_mean_, and V_max_, and principal component 2 included t_1_, t_2_, t_2_-t_1_, and T_max_. In addition, eight vectors, i.e., LT, ST, SCR, LTR, STP, LCR, V_max_, and V_mean_, were mutually aggregated with each other, showing that there was a relatively strong link between these parameters. Therefore, STD and SPD had direct effects on grain weight and yield.

## 4 Discussion

### 4.1 Effects of drought and re-watering on post-flowering dry matter accumulation and translocation

The process of dry matter accumulation and distribution in wheat can reflect the growth status of a wheat population and is an important factor in determining wheat grain yield (Zhai et al., 2022; Min et al., 2023). Suitable and sufficient water conditions are favorable for an increase in dry matter after flowering in wheat and can greatly improve the biological yield of wheat plants (Hirasawa et al., 2017; Gao et al., 2023). The results of this study showed that dry matter accumulation of aboveground organs was significantly affected by drought stress and decreased with increasing drought stress. This was due to the fact that a large amount of dry matter was retained in the nutrient organs under moderate drought stress, which largely limited the accumulation and translocation of dry matter, which was consistent with the results of Wang et al. (2022). The effects of drought stress on dry matter accumulation and transport in wheat were different at different periods. The drought treatment at the tillering stage was superior to that at the jointing stage, as shown by the fact that the dry matter accumulation of the stem sheaths, leaves, and spikes under the drought treatment at the tillering stage was higher than that of the stem sheaths, leaves, and spikes at the jointing stage for the different varieties. The reason may be that the drought treatments at the jointing stage not only inhibited the transport of wheat vascular bundles during stress but also had a weaker ability to recover after rehydration, which in turn affects the transport of assimilates and reduces the transport of dry matter to the spike (Lawlor et al., 1981). In contrast, the mild drought treatment at the tillering stage promoted the accumulation and translocation of assimilates in spring wheat after flowering, which promoted the amount of dry matter translocation and contribution to grain in later growth. This compensated for the limitation of its own growth and development caused by the mild drought environment in the early stage, and thus promoted grain weight, which was consistent with the study of Fang et al. (2024). This was confirmed by the ^13^C isotope labeling results for the T1 and CK treatments. At 72h, more ^13^C assimilates were stored in the stem sheath. The accumulation of ^13^C grain assimilates was higher at maturity, with assimilates transferred to the grain, and thus the grain distribution rate was higher than in other parts of the plant. Furthermore, the aboveground dry matter accumulation, nutrient transport rate, and contribution rate of XC6 were higher than those of XC22, indicating that dry matter accumulation and transport of drip-irrigated spring wheat varied according to variety characteristics, i.e., T1 was more effective for the more drought-tolerant XC6 wheat variety. The wheat dry matter accumulation and translocation rates were generally higher in 2023 than in 2024, which may be related to temperature changes during the wheat fertility stage. Therefore, under different periods of irrigation treatments, mild drought conditions at the tillering stage favored dry matter accumulation in all parts of the plant, increased the post-flowering translocation rate and contribution of nutrient organs, and ultimately promoted an increase in grain weight. However, further studies are needed on the genes that regulate dry matter accumulation and translocation in different drought-sensitive wheat types.

### 4.2 Effect of drought and re-watering on grain filling characteristics

In addition to being controlled by its own genetic characteristics, grain-filling characteristics were also affected by water and other factors (Wang et al., 2017; Pansarakham et al., 2018; Mu et al., 2018). Under conventional irrigation conditions, wheat thousand grain weight was positively correlated with the grouting rate (Cai et al., 2022), and Islam et al. (2021) found that under post-flowering drought stress, accelerated senescence of wheat flag leaves and the shortening of grain grouting duration resulted in lighter grain weights and lower grain yields. In a study of the effects of reduced irrigation on the filling characteristics of winter wheat kernels, Sheng et al. (2022) found that the appropriate amount of irrigation not only prolonged the duration of irrigation but also increased the maximum filling rate, which was beneficial for high grain weight. The results of this experiment found that moderate drought conditions prolonged the duration of days of wheat grain filling, increased the average and maximum filling rate, and thus increased grain weight. In this study, V_mean_ and V_max_ decreased with increasing drought severity. XC6 had 8.86% and 11.71% higher V_mean_ and V_max_ under the T1 treatment on average compared to the T2 treatment, while XC22 exhibited 7.80% and 10.47% higher values on average, respectively. Similarly, under the J1 treatment, the V_mean_ and V_max_ of XC6 were on average 12.73% and 16.58% higher than the J2 treatment, with XC22 showing 11.98% and 15.29% increases on average. The severe drought treatments reduced V_mean_ and V_max_ while prolonging T_max_, ultimately decreasing seed grain weight. These findings suggest that moderate drought conditions can enhance grain weight by increasing the filling rate and appropriately extending the rapid accumulation duration. The t_2_-t_1_, V_mean_, and V_max_ of XC6 were higher than that of XC22 under the T1 treatment, indicating that XC6 was more favorable for wheat grain filling. Further analysis found that there was a significant correlation between grain weight and STD, LTD, SPD, ST, STR, SCR, LT, LTR, LCR, t_1_, t_2,_ V_mean_, and V_max_, indicating that there was a significant correlation between each index of the plot experiment and the soil column cultivation experiment under the same water treatment, and that the pattern of change of the indexes of the soil column cultivation experiment reflected the pattern of change of the indexes of the plot experiment to a certain extent. This study showed some different results compared to the study of Yang et al. (2004). This may be due to differences in varieties and cultivation practices, among others. For high-yield wheat cultivation in Xinjiang, mild drought conditions at the tillering stage effectively promotes wheat grain filling, with drought-tolerant wheat (XC6) showing greater efficacy.

### 4.3 Effect of drought and re-watering on yield and its compositions

Moderate drought stress for a certain period of time is beneficial to crop yield, and after the stress is lifted, plant growth shows compensatory effects to make up for the reduced material accumulation during the stress period (Niu et al., 2018; Gao et al., 2024). When subjected to drought stress, crop yield, number of grains, and weight per grain were significantly affected (Rezaei et al., 2023; Mahrookashani et al., 2017). Saeidi et al. (2017) found that drought affects wheat thousand grain weight and grain yield. In this study, the mild drought treatment at the tillering stage reduced the number of spikes but increased the grain per spike and thousand grain weight, which in turn increased the wheat yield. Drought stress causes different levels of damage to different drought-tolerant varieties, and the recovery ability of different varieties is closely related to their drought tolerance (Abid et al., 2016c). This study showed that the yield of the drought-tolerant variety (XC6) could result from compensatory or supercompensatory effects under both the T1 and J1 treatments, whereas the drought-sensitive variety (XC22) only recovered to the CK level under the T1 treatment. Drought stress significantly affected the level of wheat yield and the differences between the varieties were significant. In this experiment, the yield of both varieties reached a maximum value under the T1 treatment. This indicates that the mild drought conditions at the tillering stage in the pre-growth period of wheat improved dry matter accumulation and grain filling after re-watering, and increased grain per spike and thousand grain weight to compensate for the yield loss caused by the decline in the number of spikes, and ultimately achieved higher yields with high efficiency and water conservation. Thus, for spring wheat, maintaining 60%-65% field capacity at tillering stage significantly increases the yield compensation effect.

## 5 Conclusions

Mild drought conditions at the tillering stage can enhance the aboveground dry matter accumulation, subsequently increasing the contribution of assimilates to the grain. Furthermore, the increased filling rate ultimately contributed to increased grain weight at maturity. The drought-tolerant wheat variety (XC6) demonstrated superior performance compared to the drought-sensitive wheat variety (XC22). Therefore, the use of a drought-tolerant wheat variety (XC6) and mild drought conditions (60%-65% FC) at the tillering stage can effectively promote wheat dry matter accumulation and translocation, promote grain filling, and ultimately increase wheat grain weight and yield.

## Data Availability

The raw data supporting the conclusions of this article will be made available by the authors, without undue reservation.
